# Profiling the Tumour Immune Microenvironment in Pancreatic Neuroendocrine Neoplasms with Multispectral Imaging Indicates Distinct Subpopulation Characteristics Concordant with WHO 2017 Classification

**DOI:** 10.1038/s41598-018-31383-9

**Published:** 2018-09-03

**Authors:** Daigoro Takahashi, Motohiro Kojima, Toshihiro Suzuki, Motokazu Sugimoto, Shin Kobayashi, Shinichiro Takahashi, Masaru Konishi, Naoto Gotohda, Masafumi Ikeda, Tetsuya Nakatsura, Atsushi Ochiai, Masato Nagino

**Affiliations:** 1 Division of Hepatobiliary and Pancreatic Surgery, National Cancer Center Hospital East, Kashiwa, Japan; 2 Division of Surgical Oncology, Department of Surgery, Nagoya University Graduate School of Medicine, Nagoya, Japan; 3 Division of Pathology, Exploratory Oncology Research & Clinical Trial Center, National Cancer Center, Kashiwa, Japan; 4 Division of Cancer Immunotherapy, Exploratory Oncology Research & Clinical Trial Center, National Cancer Center, Kashiwa, Japan; 5 Department of Hepatobiliary and Pancreatic Oncology, National Cancer Center Hospital East, Kashiwa, Japan

## Abstract

We successfully determined the difference of immune microenvironments between pNENs and pancreatic ductal adenocarcinomas (PDACs), and the histology-dependent variability among pNENs using multispectral fluorescent imaging system. Tumour tissue samples including 52 pNENs and 18 PDACs were investigated. The tumour-infiltrating lymphocytes (TILs), their PD-1 and PD-L1 expression in the pNENs were comprehensively and quantitatively analysed and were subsequently compared with those in PDACs. A principal component analysis revealed that the tissue immune profile is related to tumour histology, with distinct groups being observed for NETs, NECs, and PDACs. While NECs and some PDACs had hot immune microenvironments with abundant TILs, NETs had a cold immune microenvironment with few TILs. Moreover, in NETs, the numbers of intraepithelial PD-1^high^ T cells and PD-L1^high^ Type-II macrophages were elevated according to the grade. Univariate analysis revealed that lymph node metastasis, grade, stage, PD-1^high^ T cells, and PD-L1^high^ Type-II macrophages were predictors for recurrence-free survival (RFS), while grade and PD-1^high^ T cells were prognostic factors for overall survival (OS). We also showed that PD-1^high^ T cells and PD-L1^high^ Type-II macrophages were associated with worse outcome in pNENs. Our results support the WHO 2017 tumour classification criteria, which distinguish between G3 NETs and NECs.

## Introduction

Pancreatic neuroendocrine neoplasms (pNENs) are a rare type of pancreatic tumour, accounting for 2–5% of pancreatic cancers, and their incidence is estimated to be less than 1 case per 100,000 persons each year^1,2^. However, autopsy studies that account for small tumours measuring less than 5 mm suggest a much higher incidence^3^. Thus, studying pNENs is clinically important due to the growing incidence, distinct biological properties, and need for new therapeutic strategies. In 2017, the World Health Organization (WHO) classified pNENs not only by their mitotic count and the proliferation index with Ki-67 expression, but also by their morphological features. These include the presence of a well-differentiated pancreatic neuroendocrine tumour (NET) rated G1, G2, or G3 and poorly differentiated neuroendocrine carcinoma (NEC), dividing NEC G3 in WHO 2010 into NET G3 and NEC in WHO 2017. It is important to note that the tumours defined as NECs by the WHO 2017 classification could be more sensitive to platinum-based chemotherapy than those categorized as NET G3 tumours^4^, but the response of pNENs to this and other treatments has not been comprehensively investigated and additional therapeutic strategies are still needed.

Recently, the successful use of immune checkpoint inhibitors has been a big breakthrough in the development of cancer immunotherapy. The clinical development of inhibitors against programmed cell death 1 (PD-1) and its ligand (PD-L1) as anticancer agents has broadened since the approval of pembrolizumab for the treatment of advanced melanoma in September 2014^5,6^, and currently, immune checkpoint inhibitors targeting the PD-1/PD-L1 axis are approved for the treatment of several other malignancies^7^. Unfortunately, several immune checkpoint inhibitors have failed to improve survival in pancreatic ductal adenocarcinoma (PDAC) patients^8,9^, since PDAC often have a suppressive immunological status compared with other solid cancers^10^. To date, the tumour immune microenvironment has not been well characterized in pNENs. A comprehensive, comparative analysis of the tumour immune microenvironment in pNENs and PDACs could help estimate the therapeutic response to immune inhibitors, ultimately highlighting the potential efficacy of these treatments and enhancing patient care.

In the present study, we investigated the immune profile of pNENs and compared it with that of PDACs, and evaluated the histology-dependent heterogeneity of the immune profile in pNEN tumours using a multispectral imaging system. The utility of multispectral fluorescent imaging systems has been reported previously^11^. This imaging system enables staining with up to 6 antibodies and nuclei staining on a single slide. Furthermore, quantitative and topological information can be obtained, providing comprehensive and objective information regarding complex human cancer tissue. To our knowledge, this is the first study to utilize this technique to study the immune profiles of pNENs and PDACs in parallel.

## Results

### Clinicopathological characteristics

Data on patient characteristics were collected for all 70 patients (52 pNEN and 18 PDAC) and are summarized in Table 1. Twenty-nine patients were females and 23 were males, with a median age of 56 years (ranging from 31 to 77 years). The sex distribution for the PDAC cases was 10 females and 8 males, with a median age of 69.5 years (ranging from 46 to 77 years). Notably, 17 pNEN cases and 9 PDAC cases had regional lymph node metastasis. Among the 52 pNEN cases, 20 were localized in the pancreas head, 30 were in the pancreas body to tail, and 2 were in the entire pancreas. Only 6 pNEN patients had been treated with enucleation. Further, 16 pNEN patients and 10 PDAC patients had undergone pancreatoduodenectomy, while 29 pNEN and 6 PDAC had undergone distal pancreatectomy. Only 1 pNEN patient and 2 PDAC patients had been treated with total pancreatectomy. According to the WHO 2017 classification, 32 of the pNEN cases were graded as G1, 15 as G2, 3 as G3 NET, and 2 as NEC.

**Table 1:** Patient characteristics for the 52 pNEN and 18 PDAC patients included in this study.

Variables	pNEN (n = 52)	PDAC (n = 18)	*p* value
**Patient characteristics**			
Age (median, range)	56.0, 31–77	69.5, 46–77	0.001
≤60	32	4	0.004
60<	20	14	
Gender			
Female	29	10	0.987
Male	23	8	
Tumour size (median, range) (mm)	19.5, 6–92	25.5, 10–120	0.155
≤20	27	5	0.076
20<	25	13	
Lymph node metastasis	17	9	0.190
lymphatic invasion (ly)	16	12	0.007
vascular invasion (v)	29	17	0.003
perineural invasion (ne)	18	18	<0.001
Tumour Location			
Ph	20	10	0.387
Pbt	30	7	
Phbt	2	1	
Surgical Procedure			
Enucleation	6	0	0.041
Pancreatoduodenectomy	16	10	
Distal pancreatectomy	29	6	
Total pancreatectomy	1	2	
Grade (WHO 2017)			
G1	32		
G2	15		
G3	3		
NEC	2		
pStage (7th-UICC)			
IA	17	0	
IB	10	1	
IIA	8	7	
IIB	14	9	
III	0	1	
IV	3	0	
pStage (ENETS)			
I	18	3	
IIA	11	3	
IIB	6	3	
IIIA	0	0	
IIIB	14	9	
IV	3	0	

Pearson’s chi-squared tests were used to compare age, sex, tumour size, lymph node metastasis, lymphatic invasion, vascular invasion, perineural invasion, tumour location, and surgical procedure. Mann-Whitney *U* tests were used to compare age and tumour size.

### Comprehensive analysis of the TILs in pNEN

In our analysis, 52 pNENs were dichotomized into low and high subgroups according to the amount of each immune cell type (cut off median). We then analysed the correlation between each clinicopathological feature and the presence of TILs in both the epithelial and stromal regions separately (Table 2). TILs in the epithelial region, e.g. CD4+ T cells, CD204+ macrophage, and PD-1/PD-L1 expression, appeared to be more closely associated with the WHO grade and European Neuroendocrine Tumour Society (ENETS) stage. Thus, we focused on the epithelial region for the subsequent analyses in this study.

**Table 2:** Comparison of immune cell characteristics and grade/stage in the epithelial (top panel) and stromal (bottom panel) regions.

variables	CD3 low	CD3 high	*p* value	CD3+/CD4 low	CD3+/CD4 high	*p* value	CD3+/CD8 low	CD3+/CD8 high	*p* value	CD20 low	CD20 high	*p* value
WHO Grade (G1, 2/G3, NEC)	24/2	23/3	0.638	26/0	21/5	0.019	24/2	23/3	0.638	24/2	23/3	0.638
ENETS Stage (I, IIA/IIB-IV)	14/12	15/11	0.780	18/8	11/15	0.051	13/13	16/10	0.402	16/10	13/13	0.402
**variables**	**CD204 low**	**CD204 high**	*p* **value**	**CD3+/PD-1 low**	**CD3+/PD-1 high**	*p* **value**	**CD204+/PD-L1 low**	**CD204+/PD-L1 high**	*p* **value**			
WHO Grade (G1, 2/G3, NEC)	26/0	21/5	0.019	26/0	21/5	0.019	26/0	21/5	0.019			
ENETS Stage (I, IIA/IIB-IV)	17/9	12/14	0.163	19/7	10/16	0.012	19/7	10/16	0.012			
**variables**	**CD3 low**	**CD3 high**	*p* **value**	**CD3+/CD4 low**	**CD3+/CD4 high**	*p* **value**	**CD3+/CD8 low**	**CD3+/CD8 high**	*p* **value**	**CD20 low**	**CD20 high**	*p* **value**
WHO Grade (G1, 2/G3, NEC)	24/2	23/3	0.638	25/1	22/4	0.158	24/2	23/3	0.638	24/2	23/3	0.638
ENETS Stage (I, IIA/IIB-IV)	18/8	11/15	0.051	14/12	15/11	0.78	14/12	15/11	0.78	16/10	13/13	0.402
**variables**	**CD204 low**	**CD204 high**	*p* **value**	**CD3+/PD-1 low**	**CD3+/PD-1 high**	*p* **value**	**CD204+/PD-L1 low**	**CD204+/PD-L1 high**	*p* **value**			
WHO Grade (G1, 2/G3, NEC)	24/2	23/3	0.638	25/1	22/4	0.158	25/1	22/4	0.158			
ENETS Stage (I, IIA/IIB-IV)	16/10	13/13	0.402	18/8	11/15	0.051	15/11	14/12	0.78			

Pearson’s chi-squared tests were used to compare WHO grade and ENETS stage.

**Figure 1:**
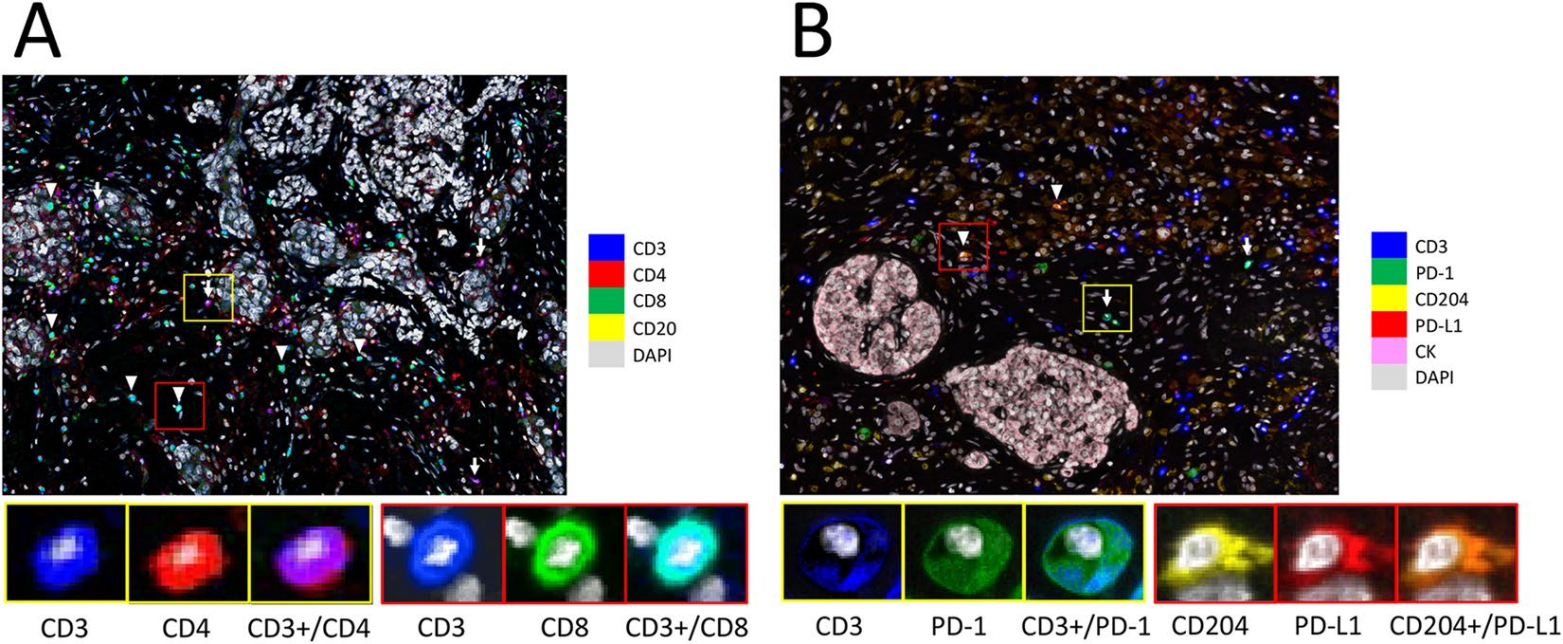
Representative images of multiplexed fluorescently-labelled NEC sections. Clear and high throughput information was used to define the immune profile. CD3+/CD4 TILs were stained purple (arrow) and CD3+/CD8 TILs were stained cyan (arrow head) (**A**), and CD3+/PD-1^high^ T cells were stained cyan (arrow) and CD204+/PD-L1^high^ macrophages were stained orange (arrow head) (**B**).

**Figure 2:**
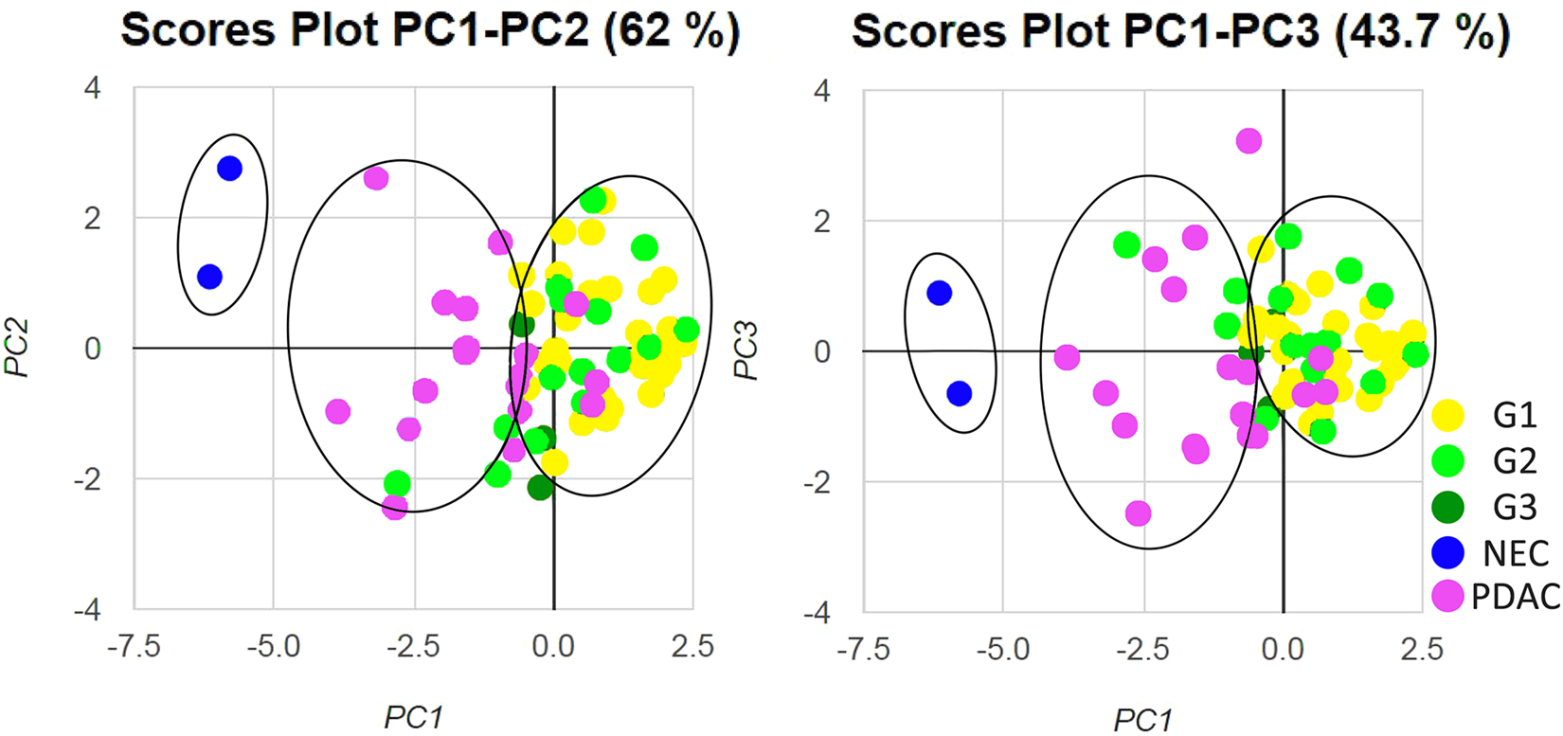
Principal component analysis of 52 pNEN and 18 PDAC patients. PCA plots of 7 variables, which includes CD3, CD4, CD8, CD20, CD204, PD-1, and PD-L1 in the epithelial area, are shown for all 70 samples. There is a clear separation of three clusters according to the histologic type of NETs, including G1/G2/G3, NECs, and PDACs.

**Figure 3:**
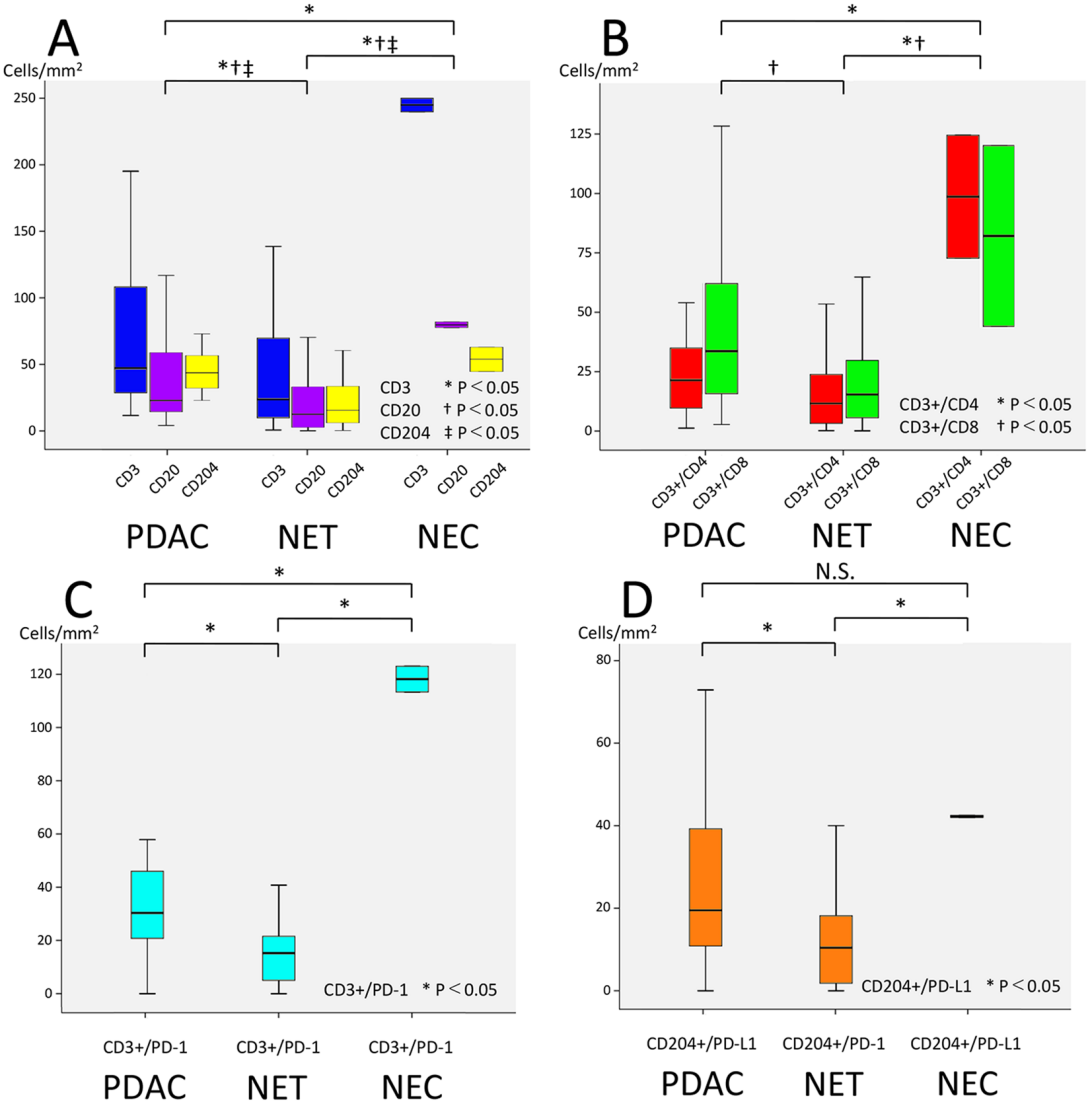
Distribution of TILs in pNENs and PDACs. The number of TILs in PDACs, NETs, and NECs was counted using the expression of CD3, CD3+/CD4, CD3+/CD8, CD20, CD204, CD3+/PD-1, and CD204+/PD-L1. P-values less than 0.05 were considered statistically significant. N.S., not significant.

**Figure 4:**
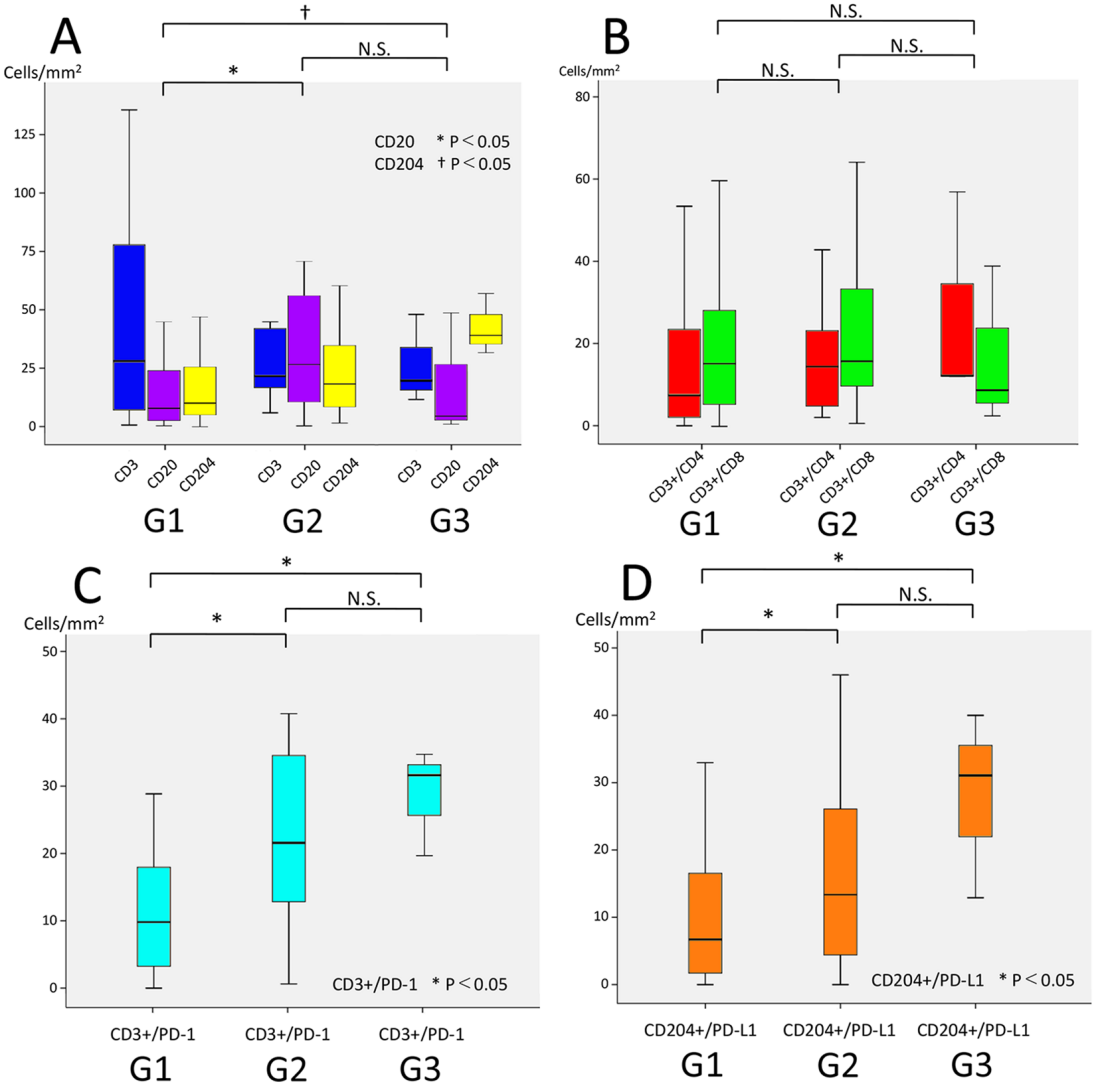
Distribution of TILs in the 50 NETs analysed in this study according to tumour grade (G1/G2/G3). The number of TILs in the 50 NETs identified in this study was counted using the expression of CD3, CD3+/CD4, CD3+/CD8, CD20, CD204, CD3+/PD-1, and CD204+/PD-L1.

Representative multiplexed fluorescently-labelled images are shown in Fig. 1, and its tissues segmented into distinct epithelial and stromal areas were shown in Supplementary Fig. S2. The profiles of TILs in all 70 pNEN and PDAC samples were plotted using PCA, and three distinct clusters, concordant with the histological types (NECs, PDACs, and G1/G2/G3 NETs), were observed (Fig. 2). These clusters indicated a correlation between the immune profile and tumour histology. In the heat map analysis, while NECs and some PDACs had hot immune microenvironments with abundant TILs, G1/G2/G3 NETs had cold immune microenvironments with few TILs (Supplementary Fig. S3). Consistent with these results, TILs and its PD-1^high^ or PD-L1^high^ subsets were less abundant in the epithelial area of NETs compared to that in the NECs and PDACs (Fig. 3). In particular, NEC showed the most intensive accumulation of CD3+/CD4, while CD3+/CD8 in PDAC and NEC were comparable. Further investigation of the TILs in the 50 patients with G1/G2/G3 NETs showed that the levels of CD3+/CD4, and CD3+/CD8 TILs were not different, but those of CD3+/PD-1^high^ and CD204+/PD-L1^high^ populations were significantly higher in higher grade NET tumours (Fig. 4). Similar to other lymphocyte subsets, CD20 TILs were less abundant in the epithelial area of NETs compared to that in the NECs and PDACs, whereas there were no tendency between the amount of CD20 TILs and grade.

### Association between TILs and patient survival

**Figure 5:**
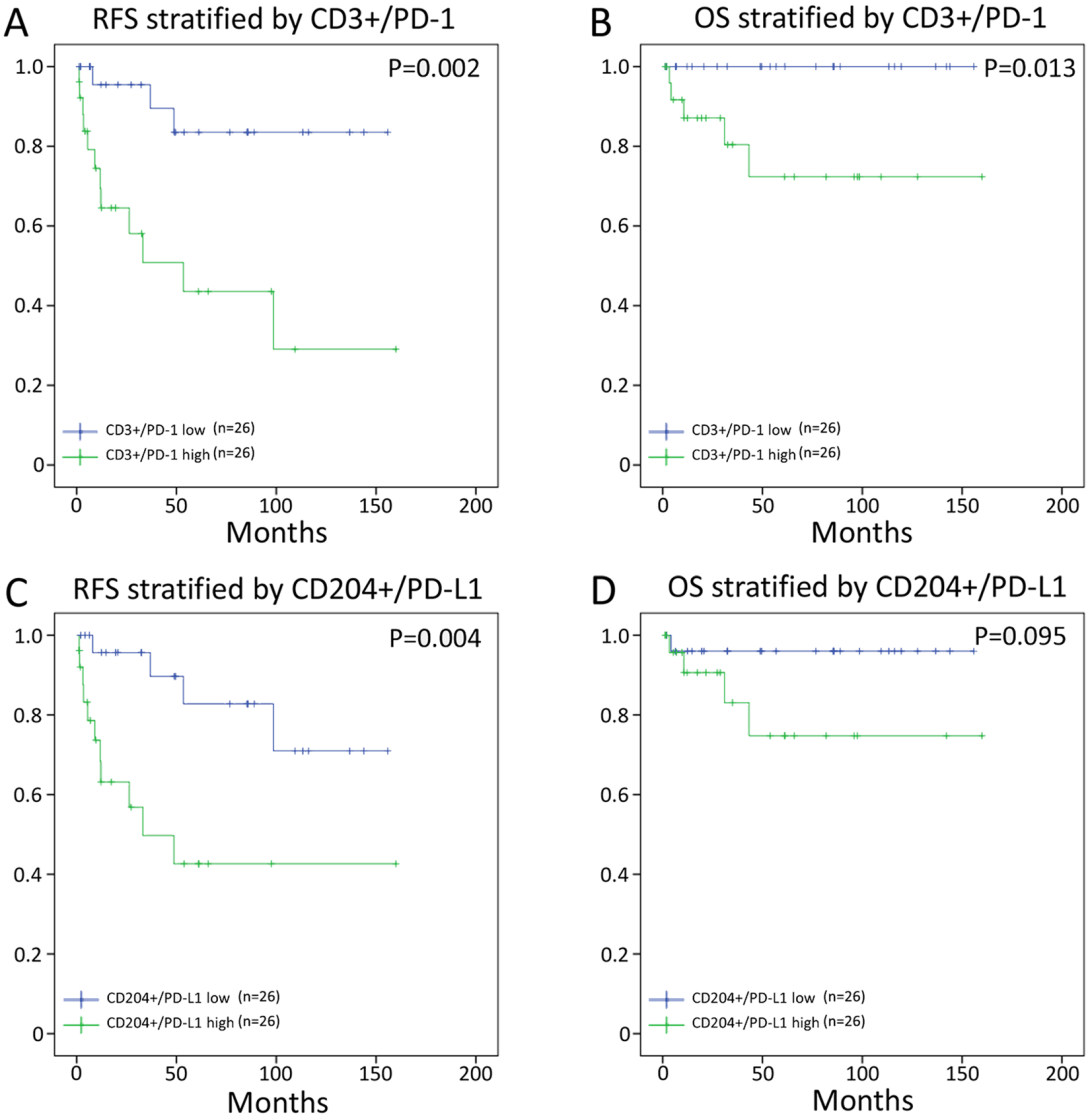
Impact of TILs on recurrence-free survival (RFS) and overall survival (OS). Kaplan-Meier survival was estimated using the CD3+/PD-1 (**A**, RFS; **B**, OS) and CD204+/PD-L1 (**C**, RFS; **D**, OS) status in the pNENs. Notably, high CD3+/PD-1 and CD204+/PD-L1 are associated with poor survival.

The results of our univariate and multivariate analyses in the 52 pNENs are shown in Tables 3 and 4, respectively, while the recurrence-free survival (RFS) and overall survival (OS) curves obtained using the Kaplan-Meier method for the pNEN patients are shown in Fig. 5. Definition of death in this study included non-disease specific death. Univariate analysis revealed that lymph node metastasis, WHO grade, ENETS stage, CD3+/PD-1^high^, and CD204+/PD-L1^high^ were predictors for RFS, while WHO grade and CD3+/PD-1^high^ were prognostic factors for OS. Multivariate analysis showed that the ENETS stage was the only independent predictive factor for RFS. Furthermore, median RFS was not reached for the CD3+/PD-1^low^ group and was 53.5 months for the CD3+/PD-1^high^ group (Fig. 5A, *p* = 0.002). Median OS was not reached for the CD3+/PD-1^low^ group and was 249.1 months for the CD3+/PD-1^high^ group (Fig. 5B, *p* = 0.013). Furthermore, median RFS was not reached for the CD204+/PD-L1^low^ group and was 33.2 months for the CD204+/PD-L1^high^ group (Fig. 5C, *p* = 0.004). Median OS was not reached for the CD204+/PD-L1^low^ group and was 249.1 months for the CD204+/PD-L1^high^ group (Fig. 5D, *p* = 0.095). Notably, the other immune cell phenotypes tested (CD3, CD3+/CD4, CD8, CD20, CD204) were not associated with clinical outcome (data not shown).

**Table 3:** Univariate and multivariate analyses of recurrence-free survival (RFS) in the 52 pNEN patients included in this study.

RFS in pNENs	univariate analysis		multivariate analysis	
variables	HR (95% CI)	p value	HR (95% CI)	*p* value
Age (60<)	1.458 (0.525–4.051)	0.467		
Sex (male)	1.257 (0.455–3.472)	0.658		
Lymph node metastasis	3.458 (1.223–9.776)	0.013	1.770 (0.518–6.061)	0.363
WHO Grade (G1, 2/G3, NEC)	6.993 (2.160–22.727)	0.001	2.179 (0.580–8.197)	0.249
ENETS Stage (I, IIA/IIB-IV)	10.821 (2.430–48.181)	<0.001	9.087 (1.374–60.085)	0.022
CD3+/PD-1 high	5.952 (1.669–21.277)	0.002	1.618 (0.333–7.874)	0.551
CD204+/PD-L1 high	4.884 (1.511–15.784)	0.004	1.751 (0.428–7.156)	0.436

The univariate analysis for age, sex, lymph node metastasis, WHO grade, ENETS stage, and for the immune profiles was performed using the log-rank test. The multivariate analysis was performed using the Cox proportional hazards model.

**Table 4:** Univariate and multivariate analyses of overall survival (OS) in the 52 pNEN patients included in this study.

OS in pNEN	univariate analysis		multivariate analysis	
variables	HR (95% CI)	*p* value	HR (95% CI)	*p* value
Age (60<)	4.396 (0.023–84.120)	0.082		
Sex (male)	1.239 (0.207–7.407)	0.814		
Lymph node metastasis	2.904 (0.484–17.416)	0.222		
WHO Grade (G1, 2/G3, NEC)	9.482 (1.496–60.092)	0.004	5.505 (0.745–40.677)	0.095
ENETS Stage (I, IIA/IIB-IV)	5.207 (0.581–46.626)	0.099		
CD3+/PD-1 high	3.458 (1.223–9.776)	0.013	3.220 (0.291–35.609)	0.340
CD3+/PD-L1 high	5.337 (0.593–48.035)	0.095		

The univariate analysis for age, sex, lymph node metastasis, WHO grade, ENETS stage, and for the immune profiles was performed using the log-rank test. The multivariate analysis was performed using the Cox proportional hazards model.

## Discussion

The histological diagnosis of pNENs is based on morphological and immunohistochemical features, including the expression of chromogranin A, synaptophysin, CD56, and Ki-67. pNENs can be subdivided into well-differentiated NETs and poorly differentiated NECs according to these histological and immunohisotchemical features. NETs can also be further subdivided into three subgroups, G1/G2/G3, according to their mitotic index and Ki-67 index. Notably, the biological features of pNENs have been well researched, and the Ki-67 proliferation index as well as CK19^12^, p27^13^, and KIT^14^ expression in the tumour cells have been identified as important predictive factors. However, these characteristics are not always a reliable reflection of treatment effectiveness or patient outcome. Recent advances in the development of cancer immunotherapy as the 4^th^ candidate for cancer treatment have rendered the evaluation of tumour immune microenvironments an informative diagnostic factor in various solid tumours. This microenvironment greatly contributes to cancer development and therapeutic resistance^15^. Unfortunately, the immune profile of pNENs is largely unknown. Therefore, in this study, we performed both quantitative and simultaneous immunohistochemical analyses using multiplexed fluorescent immunohistochemistry to evaluate the tumour immune microenvironment and to detect associations between the immune profile and the observed clinicopathological features in pNEN patients.

The use of multiplexed fluorescently-labelled images in one tissue section is a key aspect of this study as it allowed us to identify distinct subpopulations. PD-1 and PD-L1 can be expressed in other cell type than immune cell, which disturb objective expression analysis in bright field. We successfully assessed PD-1^high^ T cells and PD-L1^high^ Type-II macrophages with the combination of anti-CD3, anti-PD-1, anti-CD204, and anti- PD-L1 antibodies in one section, which were associated with poor prognosis. Although validation study of this result would be required in the future, we successfully identified clinicopathological utility of this imaging system. Furthermore, using high-throughput information of protein expression from one section enabled us to elucidate the comprehensive immune profile of pNENs. Using spatial segmentation, we classified the TILs in both the epithelium and stroma and found that TILs in the epithelium were more strongly associated with tumour grade and ENETS stage than those in the stroma. In fact, the full immune profiles in the epithelial region were strongly associated with their histological classifications (NET, NEC, or PDAC), and the NET grading could be further distinguished between G1, G2, and G3 using the CD3+/PD-1 and CD204+/PD-L1 features. These data are consistent with those of Kim *et al*.^16^, who also demonstrated similar pNEN characteristics being associated with tumour grading and poor prognosis. Furthermore, similar histology-dependent variations in the immune profile have also been reported in several other cancers^17–21^. Our results seemed to similarly reflect an association between cancer differentiation and immune profile.

It is also important to note that our results support the WHO 2017 classification, which separates the NEC G3 category in WHO 2010 into the separate G3 NET and NEC categories in 2017. This classification allows tighter clusters of characteristics to identify each group, making it possible to relate the immune profile to various morphological features. Our results suggest that NET G3 enhances immunosuppression to tumour cells compared to low-grade NET, and that NEC significantly enhances immunoresponse and subsequent immunosuppression. Moreover, our comprehensive analysis of the immune profiles of these pNEN tumours also provides information upon which future therapeutic strategies can be based. NECs are known to be histologically and genetically different from NETs^22–24^, and they require different therapeutic strategies^4^. Although platinum-based chemotherapy remains the first-line treatment, patient prognosis after treatment remains dismal. Thus, new treatment options for NEC patients based on their hot immune microenvironment need to be established. Immune checkpoint inhibitors, such as nivolumab or pembrolizumab, for example, may represent a promising new group of compounds that could be prescribed to NEC patients in the future.

In conclusion, we successfully elucidated the immune microenvironment of pNENs and highlighted the histology-dependent variability in the profile of the tumours using multispectral fluorescent imaging. To our knowledge, this is the first study to utilize this technique to study the immune profiles of pNENs and PDACs in parallel. While additional research is necessary, our data support the thesis of new WHO 2017 concept, and this comprehensive, comparative analysis of the tumour immune microenvironment in pNENs could help estimate the therapeutic response to immune inhibitors, ultimately highlighting the potential efficacy of these treatments and enhancing patient care.

## Materials and Methods

### Informed consent for study participation

All specimens were collected after obtaining written comprehensive informed consent from the patients. This study was approved by the National Cancer Ethical Review Board (reference 2016-198). All experiments were performed in accordance with relevant guidelines and regulations.

### Patients

In our analysis, formalin-fixed paraffin-embedded (FFPE) tissue specimens resected at the National Cancer Hospital East between 1994 and 2016, including 52 pNENs and 18 randomly selected PDACs without previous treatment, were studied retrospectively. pNENs were graded according to the WHO 2017 classification of tumours of endocrine organs.

### Multiplexed fluorescent immunohistochemistry

Multiplexed fluorescent immunohistochemistry was performed by the tyramide signal amplification (TSA) method using an Opal IHC kit (PerkinElmer) according to the manufacturers’ instructions. This analysis utilizes microwave treatment to remove primary and secondary antibodies, while retaining the fluorescent signal. This process is repeated until all antigens have been stained with their respective fluorophores.

Tissue sections (3 μm in thickness) were cut from each FFPE tumour specimen and then baked at 60 °C onto adhesive glass slides for 30 min before deparaffinisation. The primary antibodies used were as follows: anti-human cluster of differentiation (CD)3 (clone SP7, Abcam, Cambridge, UK; ab16669, 1:200 dilution), anti-human CD4 (clone 4B12, Novocastra, Newcastle, UK; NCL-L-CD4-368, 1:200 dilution), anti-human CD8 (clone 4B11, Novocastra; NCL-L-CD8-4B11, 1:160 dilution), anti-human CD20 (clone L26, Thermo Fisher Scientific, Rockfold, IL, USA; MA5-13141, 1:100 dilution), anti-human CD204 (SRA-E5, TransGenic Inc., Fukuoka, Japan; KT022, 1:200 dilution), anti-human PD-1 (EH33, Cell Signaling Technology, Danvers, USA; #43248S, 1:200 dilution), anti-human PD-L1 (clone E1L3N, Cell Signaling Technology; #13684P, 1:1200 dilution), and cytokeratin (clone AE1/AE3, Dako; IR053, 1:100 dilution). For staining of nuclei, spectra DAPI (PerkinElmer) was used. Tris-EDTA (pH 9) buffer was used for microwave heating before CD3, CD4, CD8, CD20, PD-1, PD-L1, and cytokeratin staining, whereas AR6 buffer (PerkinElmer) was used for CD204 staining. The antibodies were grouped into two sets for staining: set 1 (CD3, CD4, CD8, and CD20) and set 2 (CD3, CD204, PD-1, PD-L1, and cytokeratin). Opal fluorophores (PerkinElmer) were used for labelling each primary antibody. A horseradish peroxidase-labelled secondary detection system (EnVision plus, DAKO) was used as a catalyst for fluorophore-conjugated tyramide. Microwave heating (95 °C for 15 min) was performed to unmask the primary antigens and for antibody removal after each fluorescent labelling step.

### Multispectral imaging analysis and quantification

Multiplexed fluorescently labelled images (669 × 500 μm each) of the tumour margin (20 fields) and centre (20 fields) were captured with an automated imaging system (Vectra ver. 3.0, PerkinElmer). Image analysis software (InForm, PerkinElmer) was used to segment each image into cancer cell nests (intraepithelial region) and framework (stromal region) as well as to detect immune cells with specific phenotypes. Tissue segmentation and phenotype recognition were repeated until the algorithm reached the level of confidence recommended by the program supplier (at least 90% accuracy) before performing the evaluation. Infiltrating immune cells were quantified using an analytic software program (Spotfire, TIBCO, Palo Alto, CA) and then calculated per area. Using Spotfire, CD3+ population in CD4 and CD8 cells, PD-1^high^ subset in T cells, and PD-L1^high^ subset in CD204 macrophages were divided according to the fluorescence signal intensity of CD3, PD-1, and PD-L1, respectively (Supplementary Fig. S1).

### Statistical analysis

Principal component analysis (PCA) was performed using R software, version 3.3.3. All the other statistical analyses were performed using SPSS 22.0 software for Windows (SPSS, Chicago, Illinois, USA). The Mann-Whitney *U* test was used to compare continuous variables, while the chi-squared test was used to compare categorical variables. The level of significance was set at *p* < 0.05 (95% confidence). Recurrence and survival analyses were performed using the Kaplan-Meier method, starting from the date of operation. Comparisons were performed using the log-rank test, and a multivariate analysis was performed using the Cox proportional hazards model.

## Electronic supplementary material

Supplementary figure

## Data Availability

All data generated or analysed during this study are included in this published article (and its Supplementary Information files).
