# Supplementary material for: Profiling the Tumour Immune Microenvironment in Pancreatic Neuroendocrine Neoplasms with Multispectral Imaging Indicates Distinct Subpopulation Characteristics Concordant with WHO 2017 Classification
Source: Sci Rep. 2018 Sep 3;8:13166. doi: 10.1038/s41598-018-31383-9 (PMC6120899; doi:10.1038/s41598-018-31383-9)
Supplement: Supplementary file 1 — Supplementary figure [file 41598_2018_31383_MOESM1_ESM.pdf]

Original Article

Profiling the Tumour Immune Microenvironment in Pancreatic Neuroendocrine Neoplasms with Multispectral Imaging Indicates Distinct Subpopulation Characteristics Concordant with WHO 2017 Classification

Daigoro Takahashi[1, 2]; *Motohiro Kojima[3]; Toshihiro Suzuki[4]; Motokazu Sugimoto[1]; Shin Kobayashi[1]; Shinichiro Takahashi[1]; Masaru Konishi[1]; Naoto Gotohda[1]; Masafumi Ikeda[5]; Tetsuya Nakatsura[4]; Atsushi Ochiai[3]; and Masato Nagino[2]

[1] Division of Hepatobiliary and Pancreatic Surgery, National Cancer Center Hospital East, Kashiwa, Japan
[2] Division of Surgical Oncology, Department of Surgery, Nagoya University Graduate School of Medicine, Nagoya, Japan
[3] Division of Pathology, Exploratory Oncology Research & Clinical Trial Center, National Cancer Center, Kashiwa, Japan
[4] Division of Cancer Immunotherapy, Exploratory Oncology Research & Clinical Trial Center, National Cancer Center
[5] Department of Hepatobiliary and Pancreatic Oncology, National Cancer Center Hospital East, Kashiwa, Japan

Supplementary figure 1

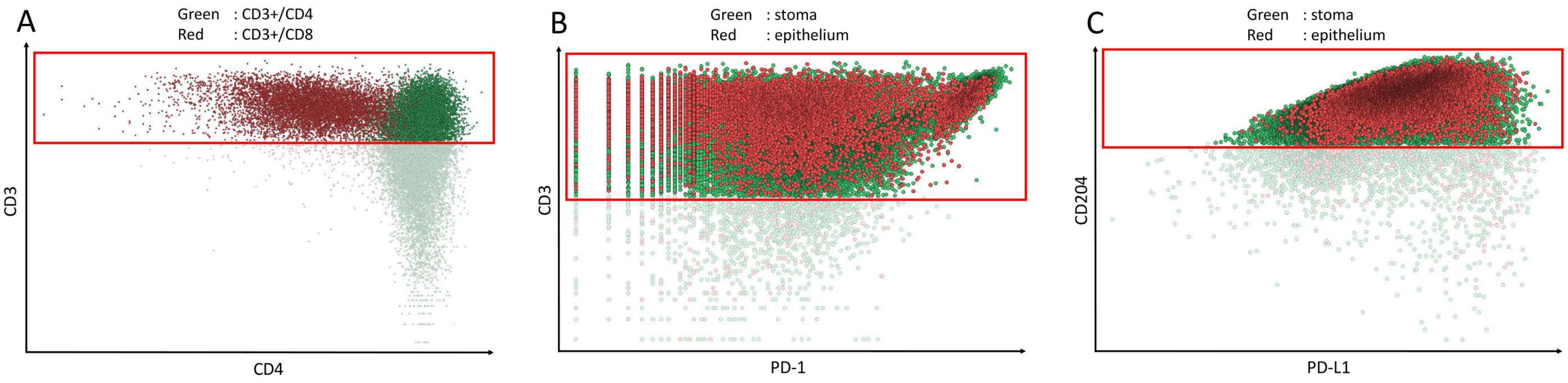

Supplementary fugure 2

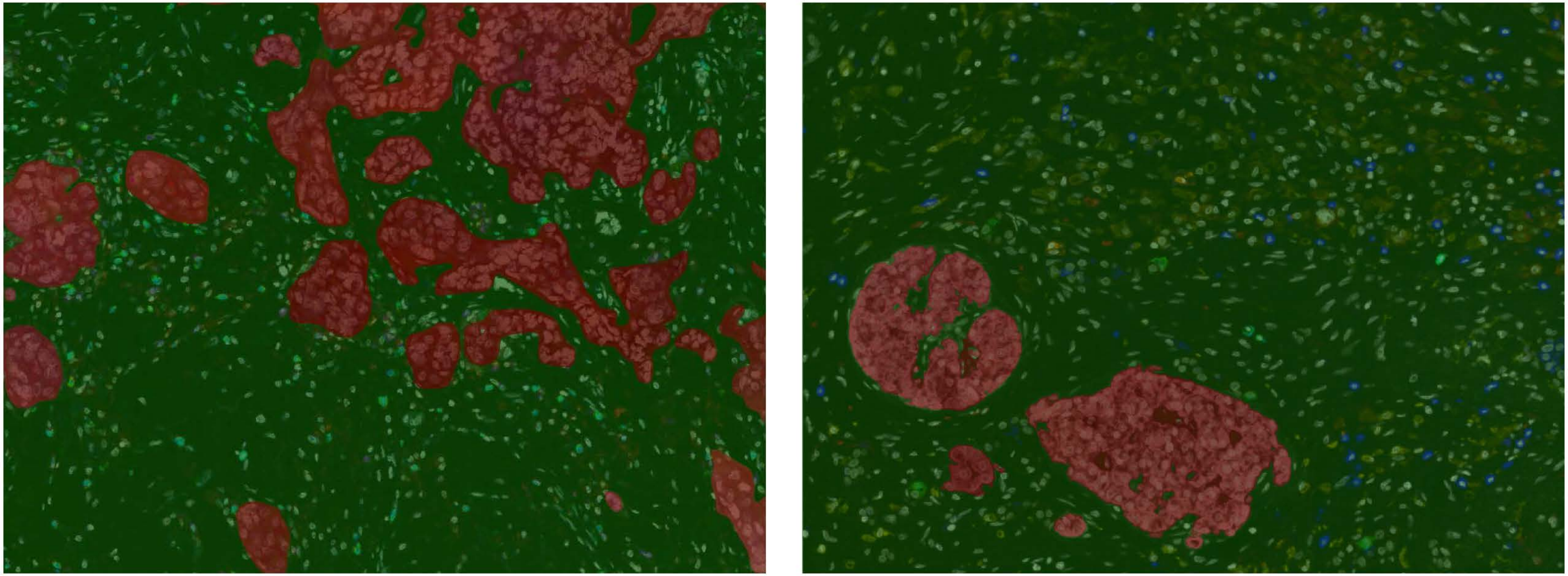

Supplementary figure 3

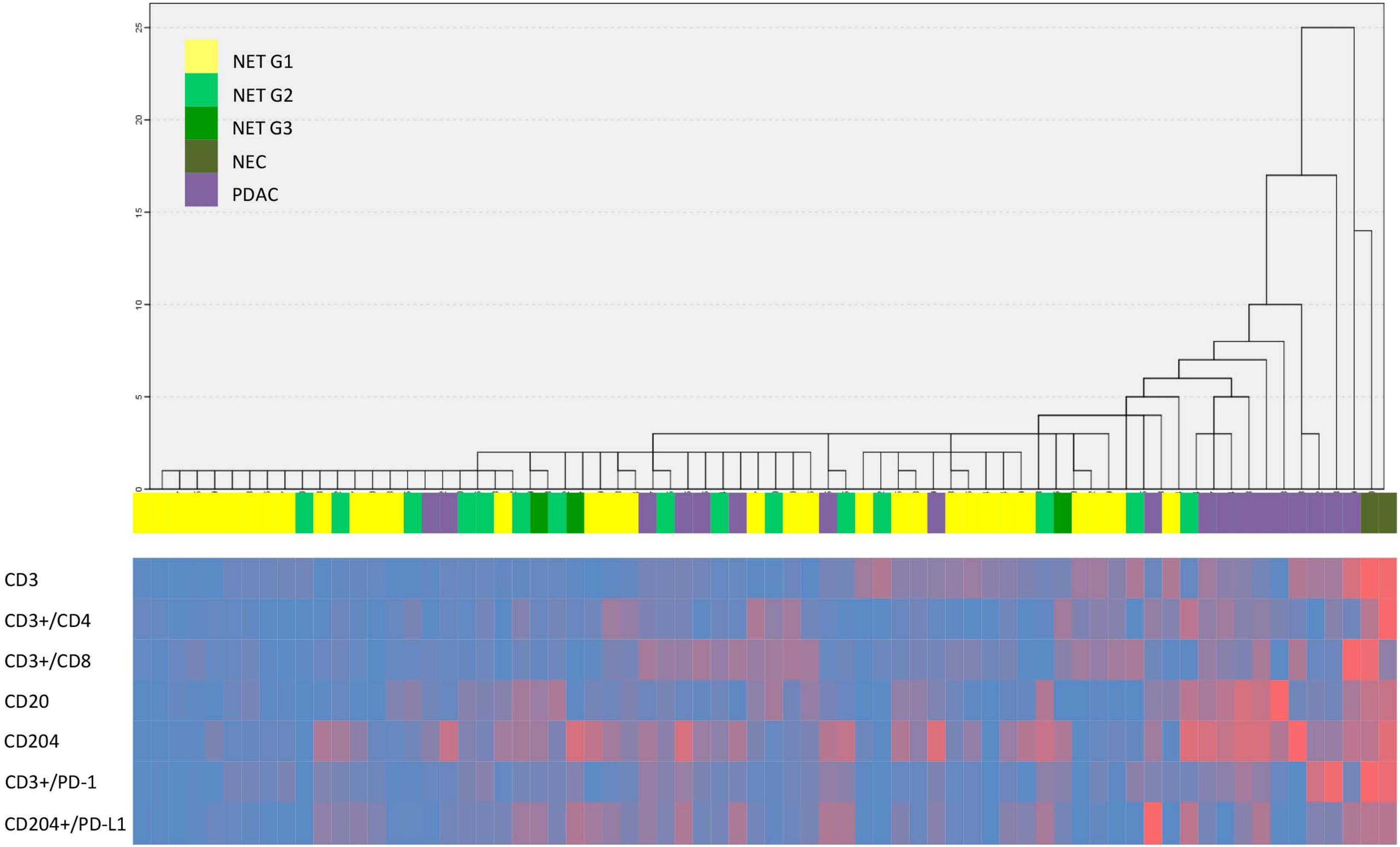

Figure S1. Selection of CD3+/CD4 TILs, CD3+/CD8 TILs, PD-1^{high} subset in T cells and PD-L1^{high} subset in CD204 macrophage. CD4 and CD8 TILs were gauged by fluorescence signal intensity of CD3 (A). PD-1^{high} subset in T cells and PD-L1^{high} subset in CD204 macrophage were shown in (B) and (C) respectively.

Figure S2. Representative images segmented into epithelial and stromal areas by imaging analysis software. Epithelial areas were shown in red and stromal areas were shown in green. The areas in Figure S2 are concordant with that in Figure 1.

Figure S3. Hierarchical cluster analysis displayed as a dendrogram and heat map of the immune profile of NETs, NECs, and PDACs. NECs and some PDACs appear to have hot immune microenvironments, while NETs have a cold immune microenvironment.
